# Femoral nerve block versus obturator nerve block for pain management after total knee replacement: A randomized controlled trial protocol

**DOI:** 10.1097/MD.0000000000021956

**Published:** 2020-09-11

**Authors:** Wei Zhang, Peng Lin, Fuheng Zhang, Ji Wang

**Affiliations:** Department of Orthopaedics, NO.971 Hospital of the People's Liberation Army Navy, Shangdong, China.

**Keywords:** femoral nerve block, obturator nerve block, pain management, protocol, total knee replacement

## Abstract

**Background:**

Several studies reported short-term analgesic efficacy of obturator nerve block (ONB), as in comparison with the femoral nerve block (FNB) in the treatment of postoperative pain after the total knee replacement (TKR). The optimal method remains under debate. The purpose of our current work is to compare the safety and efficacy of FNB and ONB for postoperative analgesia after TKR.

**Methods:**

This prospective, randomized, and controlled study was performed from January 2018 to December 2019. It was authorized via the Institutional Review Committee in NO.971 Hospital of the People's Liberation Army Navy (2019-PLAN-132).

Two hundred patients were divided randomly into 2 groups, the control group (n = 100) and study group (n = 100). The experimental group received FNB and control groups received ONB. Primary outcome included pain at different time point (Visual Analogue Scale score of anterior knee pain at rest and in motion). The Visual Analogue Scale scores were marked by patients themselves on a paper with a graduated line starting at 0 (no pain) and ending at 10 (the most painful). Opioid consumption was converted to equivalents of oral morphine uniformly for statistical analysis. Secondary outcomes included the knee range of motion, the hospital stay length as well as the postoperative complications such as pulmonary embolism and deep vein thrombosis.

**Results:**

Table 1 will show the clinical outcomes between the 2 groups.

**Conclusion:**

This trial would provide an evidence for the use of different types of peripheral nerve blocks in TKR.

## 1 Introduction

The total knee replacement (TKR) is regarded as a successful method option for treating the end-stage knee osteoarthritis, with satisfactory functional recovery, significant deformity correction effect, and outstanding postoperative pain relief.^[1,2]^ With the aging of the US population, the number of TKR is expected to obviously increase by 2030, reaching estimated 3.48 million a year.^[3,4]^ In the acute stage after surgery, TKR can lead to obvious pain, and the postoperative knee pain has become a familiar and persistent chief complaint after the TKR, leading to patient poor life quality and dissatisfaction. The reported incidence of severe postoperative knee pain after TKR ranges from 10.3% to 36.8%.^[5,6]^ Opioid is frequently used for postoperative pain management. However, it may be associated with many adverse effects, including headache, nausea, vomiting, respiratory depression, retention of urine, and constipation.^[7–9]^ Specific medical diseases related to the inadequate pain control involved myocardial infarction, coronary ischemia, venous thrombosis, and pneumonia. Effective postoperative analgesia can reduce opioid consumption and promote rehabilitation.

Several methods have been applied to reduce postoperative pain after TKR including the peripheral nerve block and the local infiltration anesthesia, intravenous analgesics as well as the epidural anesthesia.^[10–13]^ The optimal method remains under debate. Femoral nerve block (FNB) was reported to reduce postoperative pain and has increased in popularity because of its opioid sparing effects, and consistency with anticoagulatory therapy. However, some articles reported that FNB may decrease the strength of quadriceps femoris, which causes risk of fall.^[14]^ Recently, several studies reported short-term analgesic efficacy of obturator nerve block (ONB), as in comparison with the FNB in the treatment of postoperative pain after TKR. Bergeron et al^[15]^ reported that there was no significant difference between FNB and ONB in terms of functional outcomes. Currently, there is no reliable evidence to support the clinical application for pain control. The purpose of our current work is to compare the safety and efficacy of FNB and ONB for postoperative analgesia after TKR.

## 2 Methods

### 2.1 Study design

This controlled, randomized, and prospective research was performed from January 2018 to December 2019 which was performed in accordance with the SPIRIT Checklist for randomized studies. It was authorized via the Institutional Review Committee in NO.971 Hospital of the People's Liberation Army Navy (2019-PLAN-132) and then was registered in research registry (researchregistry5849).

### 2.2 Recruitment and consent

All patients participating in the TKR will be treated in our orthopedic outpatient department. The surgeon will explain the details of trial, afterward, patiently answer all the patient's questions. The patient is then presented with the written information of our trial. Each of patient received a written informed consent. Since all patients participated voluntarily, they could withdraw at any time during the trial.

### 2.3 Inclusion of exclusion criteria

The subjects in this research were 200 primary TKR patients from our Hospital. The inclusion criteria were:

(1) patients prepared for primary unilateral TKR;
(2) the age were above 50 years.

The exclusion criteria were as follows:

(1) the body mass index of the patients was more than 40 kg/m^2^;
(2) allergies or contraindications to the opioid analgesics;
(3) a history of deep vein thrombosis or pulmonary embolism 3 months before the operation.

### 2.4 Randomization and Blinding

Two hundred patients were divided randomly into 2 groups, namely, control group (n = 100) and the study group (n = 100), respectively. A table of random numbers hidden in the 1:1 ratio was computer-formed. A researcher who did not take part in the trial used the website Randomization.com to generate a random distribution sequence, which was hidden in sealed opaque sequence numbered envelopes that were allocated to investigators. The surgeons, investigator, anesthetist, and nurses were all kept blinded to allocation results.

### 2.5 Intervention of each group

All patients were given the spinal anesthesia. The surgical procedures were performed by the senior surgeon. In the process of operation, pneumatic tourniquet was utilized. An incision was made in the center of the knee and then extended to the medial side of patella. A posterior stabilized prosthesis (Attune, DePuy, Warsaw, IN) was implanted. The experimental group received FNB and control groups received ONB. Other procedures were the same: removing excess peri-patella synovium and osteophytes, trimming the patella with a narrow oscillating saw and circumferential electrocautery of the patella. Multimodal postoperative pain management of our center were administered to all the patients. Analgesic protocol: after admission, patients were given 200 mg celecoxib orally every 12 hours for preemptive analgesia until the morning of the operation day. Intraoperatively, patients were given 20 mg ropivacaine diluted with 60 mL normal saline by topical injection to the joint capsule and collateral ligaments. Intravenous patient-controlled analgesia was given after TKR.

### 2.6 Outcome measures

Post-operative clinical data were assessed by an independent senior surgeon blinded to the patient's randomization. Primary outcome included pain at different time point (Visual Analogue Scale score of anterior knee pain at rest and in motion). The Visual Analogue Scale scores were marked by patients themselves on a paper with a graduated line starting at 0 (no pain) and ending at 10 (the most painful). Opioid consumption was converted to equivalents of oral morphine uniformly for statistical analysis. Secondary outcomes included the knee range of motion, the hospital stay length as well as the postoperative complications such as pulmonary embolism and deep vein thrombosis.

### 2.7 Statistical analysis

The calculations of sample size are conducted utilizing the software of PASS 2011 (NCSS, LLC, Kaysville, UT). All the needed analyses are implemented through utilizing SPSS for Windows Version 20.0. All the data are represented with proper characteristics as median, mean, percentage as well as standard deviation. Mann–Whitney *U* test or the independent samples *t*-test were used to analyze the inter-group comparison. Chi-square detection was utilized to compare the categorical variables among the groups. The analysis of repeated measurement of the variance was applied to analyze the repeated data. A *P* < .05 was regarded the significant in statistics.

## 3 Result

Table 1 will show the clinical outcomes between the 2 groups.

**Table 1:**
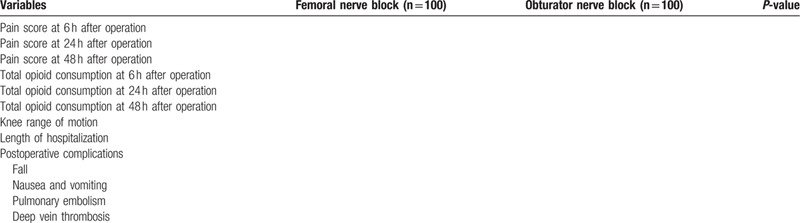
Outcome measures between femoral nerve block and obturator nerve block.

## 4 Discussion

Pain management after the TKR is still challenging, but is important due to good pain management can improve the outcomes of patient. About half of patients who undergo the TKR experience moderate to serious postoperative pain.^[16]^ The postoperative pain is caused by inflammation resulted from direct nerve injury or the tissue injury. Patients can perceive the pain through afferent pain pathway, this is the target of a variety of drugs. Direct anesthetics or the drugs that reduce the response of local hormones to injury (nonsteroidal anti-inflammatory drugs, such as ibuprofen or aspirin) can be utilized to block the activity of pain receptors, thereby reducing the activity of pain receptors. The various management programs of postoperative pain and the lack of a definitive “gold standard” indicate that there is room for improving the standard of care.^[17]^

Peripheral nerve block with continuous or single infusion of local anesthetics has been extensively utilized in TKR field. The more familiar forms of peripheral nerve block in the TKA are the sciatic nerve, obturator nerve, and the FNBs.^[18,19]^ Despite muscle weakness after surgery, there is evidence that the patients receiving the peripheral nerve block can recover faster than the patients receiving patient-controlled analgesia. Thus, we performed this protocol to compare the efficacy of FNB and ONB for pain management in patients undergoing TKR.

## 5 Conclusion

This trial would provide an evidence for the use of different types of peripheral nerve blocks in TKR.

## Author contributions

**Data curation:** Peng Lin.

**Investigation:** Fuheng Zhang.

**Methodology:** Fuheng Zhang.

**Writing – original draft:** Wei Zhang.
